# Outcomes Following Catheterization for ST-Segment Elevation Myocardial Infarction in Older Adults With Dementia

**DOI:** 10.1093/geroni/igaf122.3632

**Published:** 2025-12-31

**Authors:** Michael Nanna, Leila Agha, Ashleigh Erickson, Bangyao Sun, Raiza Rossi, Christopher Leggett, Douglas Staiger, Andrew Cohen

**Affiliations:** Yale University, New Haven, Connecticut, United States; Harvard Medical School, Boston, Massachusetts, United States; Geisel School of Medicine, Lebanon, New Hampshire, United States; Harvard Medical School, Boston, Massachusetts, United States; Yale University, Yale University, Connecticut, United States; Geisel School of Medicine, Lebanon, New Hampshire, United States; Dartmouth College, Hanover, New Hampshire, United States; Yale School of Medicine, New Haven, Connecticut, United States

## Abstract

ST-segment elevation myocardial infarction (STEMI) is a common cardiac emergency involving complete occlusion of one of the coronary arteries, for which left heart catheterization is the gold standard treatment. Older adults with Alzheimer’s disease and related dementias (ADRD) are less likely to receive this treatment, but little is known about the outcomes among those who do. We used a 100% Medicare fee-for-service sample from 2017-2022 to compare outcomes following STEMI among individuals with and without ADRD who underwent catheterization, stratified by pre-event care setting (community versus nursing home). The primary outcome was days alive at home in the year following STEMI; secondary outcomes included one-year mortality, proportion of alive days at home, and long-term care use. Differences between groups were adjusted for age, sex, and comorbidities. Among 117,318 patients, 6.3% had ADRD and 97.8% were admitted from the community. Compared to those without ADRD, community-dwelling patients with ADRD had higher one-year mortality rates (36.6% vs 16.6%; adjusted difference, 10.0%) and fewer home days (225 vs 299; adjusted difference, 37.4), yet over two-thirds spent more than 300 days at home and only 3.9% entered long-term nursing home care. Patients admitted from nursing homes had worse outcomes, regardless of ADRD status, with one-year mortality rates of 57.6% (ADRD) and 40.3% (without ADRD; adjusted difference, 17.3%). Our findings suggest that older adults with ADRD who undergo catheterization for STEMI, particularly if admitted from the community, have health outcomes that many may find acceptable. These results may help guide prognosis-informed, patient-centered decision-making.

